# Developing spatio-temporal approach to predict economic dynamics based on online news

**DOI:** 10.1038/s41598-022-20489-w

**Published:** 2022-09-28

**Authors:** Yuzhou Zhang, Hua Sun, Guang Gao, Lidan Shou, Dun Wu

**Affiliations:** 1 Popsmart Technology (Zhejiang) Co., Ltd., Ningbo, 315100 China; 2 College of Computer Science and Technology, Zhejiang University, Hangzhou, 310063 China

**Keywords:** Statistics, Computational science

## Abstract

Economic forecasting is a scientific decision-making tool, and it is one of the important basis for the government to formulate economic plans, predict the implementation of the plan, and guide the implementation of the plan. Current knowledge about the use of online news in the prediction of economic patterns in China is limited, especially considering the spatio-temporal dynamics over time. This study explored the spatio-temporal patterns of economic output values in Yinzhou, Ningbo, China between 2018 and 2021, and proposed generalized linear model (GLM) and Geographically weighted regression (GWR) model to predict the dynamics using online news data. The results indicated that there were spatio-temporal variations in the economic dynamics in the study area. The online news showed a great potential to predict economic dynamics, with better performance in the GWR model. The findings suggested online news combining with spatio-temporal approach can better forecast economic dynamics, which can be seen as a pre-requisite for developing an online news-based surveillance system The advanced spatio-temporal analysis enables governments to garner insights about the patterns of economic dynamics over time, which may enhance the ability of government to formulate economic plans and to predict the implementation of the plan. The proposed model may be extended to greater geographic area to validate such approach.

## Introduction

Economic forecasts are based on statistical data and economic information, starting from the status quo and laws of economic phenomena, and using scientific methods to predict the future development prospects of the economy. Economic forecasting is a scientific decision-making tool, and it is one of the important basis for the government to formulate economic plans, predict the implementation of the plan, and guide the implementation of the plan. Economic forecasting generally uses two types of subdivision methods: statistical analysis and mathematical models, which are adaptive filtering^1^, time-series forecasting^2^, trend curve forecasting models^3^, regression forecasting methods^4^, grey forecasting models^5^, Markov forecasting methods^6^, etc. There were many attempts to improve the accuracy of economic predictions using several economic indicators. For instance, Abberger and Wohlrabe applied business surveys data to forecast the economic growth^7^, and Hüfner and Schröder used the ifo and ZEW indicators to develop their predicting models^8^.

Since the age of internet, big data has been noticed as a novel data source to predict the economic activities. A previous study in the USA indicated that the number of online news with certain queries can forecast US private consumption^9^. In addition, several previous studies focused on the forecast of stock market using internet search engine data. Bordino et al. applied the search volume from Yahoo! As a predictor to predict stock market volumes^10^. Similarly, Kholodilin et al. improved the forecasts of US private consumption using the search queries data from Google^11^.

Advances in spatio-temporal analysis enables governments to garner insights about the patterns of economic dynamics, spatial clusters, and trends over time. However, current knowledge about the use of online news in the prediction of economic patterns in China is limited, especially considering the spatio-temporal dynamics over time. This study aims to examine the variations of economic patterns in Ningbo city, China at both space and time, and predict such dynamics using online news data.

## Methods

### Data collection

The annual economic output value data between 2018 and 2021 were collected from the Yinzhou Economic Statistics Platform, which including 1180 manufacture enterprises in Yinzhou, Ningbo, China in the study. We applied this dataset because of the high proportion of annual output value form these enterprises (over 80%). The included enterprises were separated by industry, including high-level equipment, key fundamental part, motorcar, fashion and clothes, new materials, intelligent home electrics, new service and others.

The annual online news data for each enterprise for the same period were collected from the Yinzhou Economic Statistics Platform, which including the number of positive and negative online news for each enterprise from several main online news websites in China (Table 1). The Platform used the name of each enterprise as the key word to collected the online news data from the targeted websites.

**Table 1:** The online news data source used in the study.

Name	Website
Baidu news	https://news.baidu.com/
Jinritoutiao	https://www.toutiao.com/
Fengguangwang	https://www.ifeng.com/
Sina News	https://news.sina.com.cn/
Souhu News	http://news.sohu.com/
Wangyi News	https://news.163.com/

In the study, we have considered the influence of policy on the economic dynamics, which mainly included the development and restriction policies announced by governments. As a result, we have transformed the policy dataset to numeric dataset (a development policy: 1, a restriction policy: − 1), which is the yearly total number of the polices in the proposed models to control this possible confounder.

## Data analysis

### Spatio-temporal cluster analysis

In order to determine the spatial clustering of economic output value, as well as online news data over time, we carried out statistics on those variables and conducted the spatial autocorrelation analysis by ArcGIS 10.3 software (https://www.esri.com/, Esri Inc, Redlands, CA, USA). Moran's I index was used to examine whether the variables have spatial autocorrelation. The value range of the Moran index I is [-1, 1], standardized statistic Z(I) was used to test statistical significance. When Moran’s I > 0 and Z > 1.96, P < 0.05, it indicates that the variable is clustered and there are aggregation areas for observed high or low values. When Moran’s I < 0 and Z < − 1.96, P < 0.05, This indicates that the cases are discretely distributed; when Moran's I = 0 and the value of Z is between 1.96 and − 1.96, P ≥ 0.05, indicating that the spatial distribution of cases may be random^12^. The Moran's I reflects the overall spatial correlation between areas, which can be expressed as below:

$$ I_{i} = \frac{{{{z}}_{i} - \overline{{{z}}}}}{{\sigma^{2} }}\sum\limits_{j = 1,j \ne i}^{n} {\left[ {{{w}}_{ij} \left( {{{z}}_{j} - \overline{{{z}}}} \right)} \right]} $$

where I_i_ represents the Moran's I coefficient; $z_{i}$ is the value of the variable (the annual economic output value, the change in annual economic output value, the annual number of positive online news, the percentage of positive online news) at the location i; $\overline{z}$ is the mean value of the variable with the sample number of n; z_j_ is the value at all the other locations (where j ≠ i); σ^2^ is the variance of z, w_ij_ is the spatial weight which can be represented based on a distance of weighting between z_i_ and z_j_.

To performed the spatial-related analysis, we transformed the point-based data to hexagon-based data, with the mean value of included point-based data for each hexagon, which can generate adjacent regions across the study site.

#### Generalized linear model (GLM)

The generalized linear models (GLM) extend the general linear model so that the dependent variable is linearly related to the factors and covariates through the specified link function^13^. In addition, the model allows the dependent variable to have a non-normal distribution^13^. This study developed GLM to quantify the interactive relationship between the dynamics of economic output values and the patterns of the number of online news over time across the study area. Multicollinearity among internal drivers was checked and avoid through checking Spearman correlation analysis and variance inflation factors (VIF). Only one of the highly-correlated drivers (VIF > 5) was included in the GLM^14^. A negative binomial distribution was assumed to allow over-dispersion^15^. The developed GLM as following:

$$\mathit{log}\left[E\left(Y\right)\right]={\beta }_{0}+{\beta }_{1}\left({V}_{1}\right)+{\beta }_{2}\left({V}_{2}\right)+{\beta }_{3}\left({V}_{3}\right)+\dots +e$$

where *E(Y)* represents the expected annual economic output value or the change of the values between years of each generated hexagon area; *β*_*0*_ represents the intercept; *β*_*1*_* (V*_*1*_*)* denotes the corresponding regression coefficient of the yearly number of positive online news for the area; *β*_*2*_* (V*_*2*_*)* represents the coefficient of the percentage of positive online news for the region; *β*_*3*_* (V*_*3*_*)* denotes the coefficient of the yearly number of industry policy of the area; and *e* represents the error.

#### Geographically weighted regression (GWR) model

To consider the spatial correlation and heterogeneity in dataset by space unit, geographically weighted regression (GWR) model was developed. GWR model can perform advanced local regression for each spatial unit, and then estimate the regression coefficients for each region.

GWR model embeds the spatial location in an ordinary linear regression (OLR) model to detect the spatial non-stationarity of things^16^. GWR model based on geographically weighted regression detects the spatiotemporal non-stationary characteristics of things and improves the multi-dimensional analysis ability of spatiotemporal data, which has important theoretical and practical significance. Its formula below was applied^17^:

$$ y_{i}  = \beta _{0} \left( {u_{i} ,v_{i} } \right) + \sum\limits_{{i = 1}}^{j} {\beta _{{1k}} (u_{i} ,v_{i} )X_{{ik}}  + \varepsilon _{i} }  $$

In the equation, *y*_*i*_ is the fitted value of the annual economic output value or the change of the values between years of each generated hexagon area *i*; *X*_*ik*_ (1,…4) is the value of the k-th independent variable, including the annual number of online news for the area, the annual number of positive online news for the region, the annual number of negative online news of the area; the percentage of positive online news for the region; *β*_*1k*_ is the k-th local regression parameter, a function of geographic location; *(u*_*i*_*, v*_*i*_*)* denotes the geographical coordinate of the center of a sample spatial unit, *β*_*0*_* (u*_*i*_*, v*_*i*_*)* represents the constant term estimate; ε is the random error term, with the mean value of 0 under an independent normal distribution.

In the GWR model, the coefficients of the estimated variables are adjusted by their spatial position changes. The estimated coefficients of parameters can be expressed as bellowing after locally weighting neighborhood locations:

$${\beta }_{k}({u}_{i},{\vartheta }_{i})=({X}^{T}W({u}_{i},{\vartheta }_{i})X{)}^{-1}{X}^{T}W({u}_{i},{\vartheta }_{i})Y$$

where *β*_*k*_*(u*_*i*_*, v*_*i*_*)* is the regression coefficient of the i-th sample, *W(u*_*i*_*, v*_*i*_*)* is the weight matrix after the geometric weighted regression of the position i. This was used to adjust the effect of observed values at different spatial unit on the overall regression estimates. The optimal bandwidth was selected by the smallest Akaike’s Information Criterion (AIC) value and standard residual in the model.

In the study, we have divided the data into two data sets: data from 2018 to 2019 was used as a training dataset to construct models and data from 2020 to 2021 was used as a test data set to validate the models^18–20^. Moreover, AIC and the stationary R square (R^2^) were used to examine the goodness-of-fit of the model in training period, and also can compare the fitness performance between the GLM and GWR models. Additionally, the predictive performance of GWR models was evaluated by three metrics in validation period: Pearson correlation, Root Mean Squared Error (RMSE) and Maximum Absolute Percent Error (MAPE), which were widely used in forecasting studies to indicate the discrepancies between observed and predicted values^18–21^.

## Results

### Spatio-temporal distribution of economic output value

**Figure 1:**
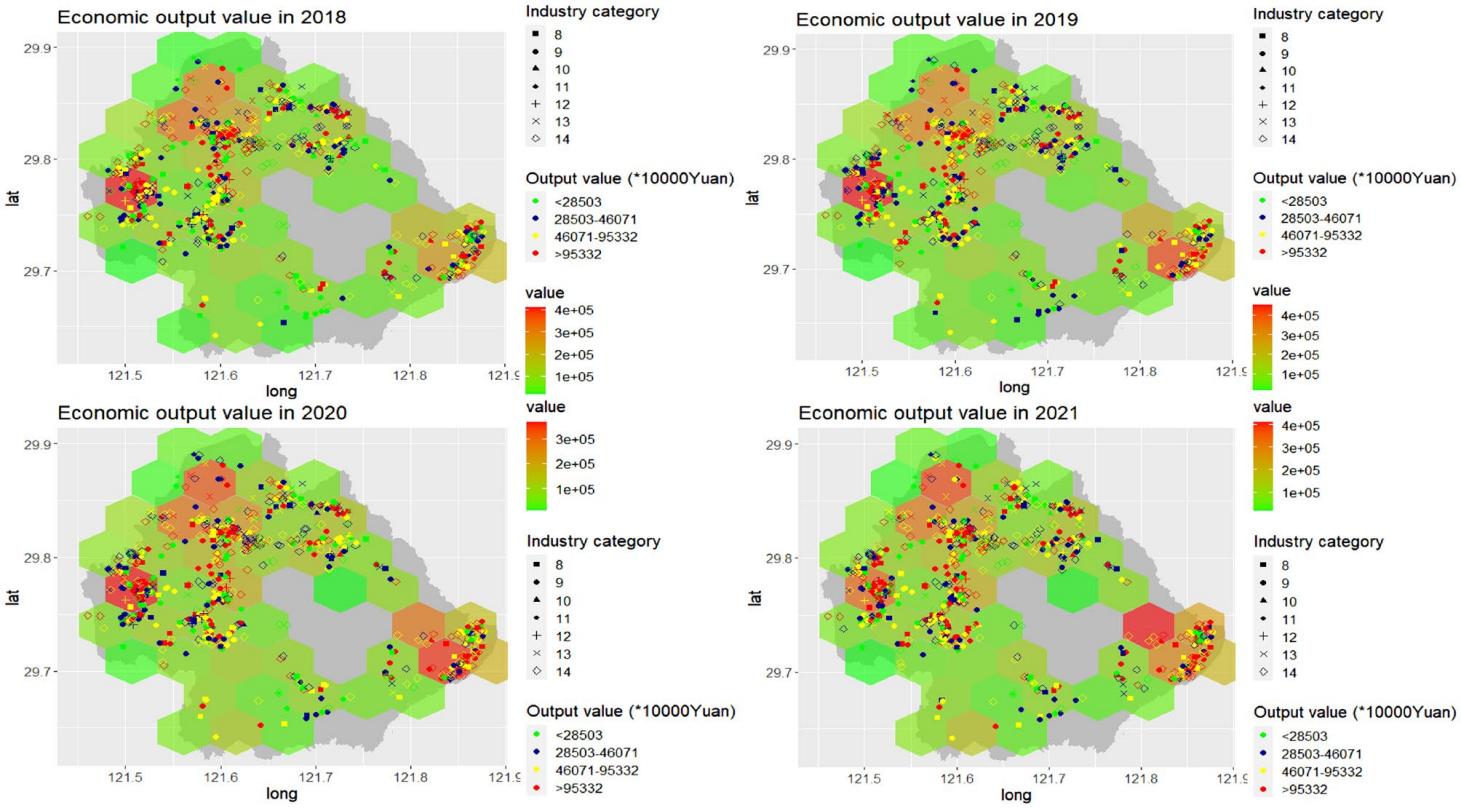
The spatio-temporal distribution of economic output value in Yinzhou, 2018–2021 (Industry category: 8 (motorcar), 9 (high-level equipment), 10 (new materials), 11 (intelligent home electrics), 12 (key fundamental part), 13 (fashion and clothes), 14 (new service and others)).

Figure 1 showed the spatial distribution of annual economic output value and online news number from 2018 to 2021. The minimum economic output value was 1,032, and the maximum was 19,845,026, with the mean of 155,378 in 2018. The smallest value in 2019 was 13,684, and the biggest one was 20,352,154, with the mean of 173,630. In 2020, the minimum value was 10,928, and the maximum one was 12,972,880, with the mean of 162,638. The minimum economic output value was 10,595 in 2021, and the maximum was 18,565,926, with the mean of 164,944. Additionally, it can be seen that the agglomeration of the economic output value is obvious in space during the study period. The regions with relatively higher economic output value were found to be concentrated in the northwest and southeast part in Yinzhou.

### The variation of economic dynamics in space and time

**Figure 2:**
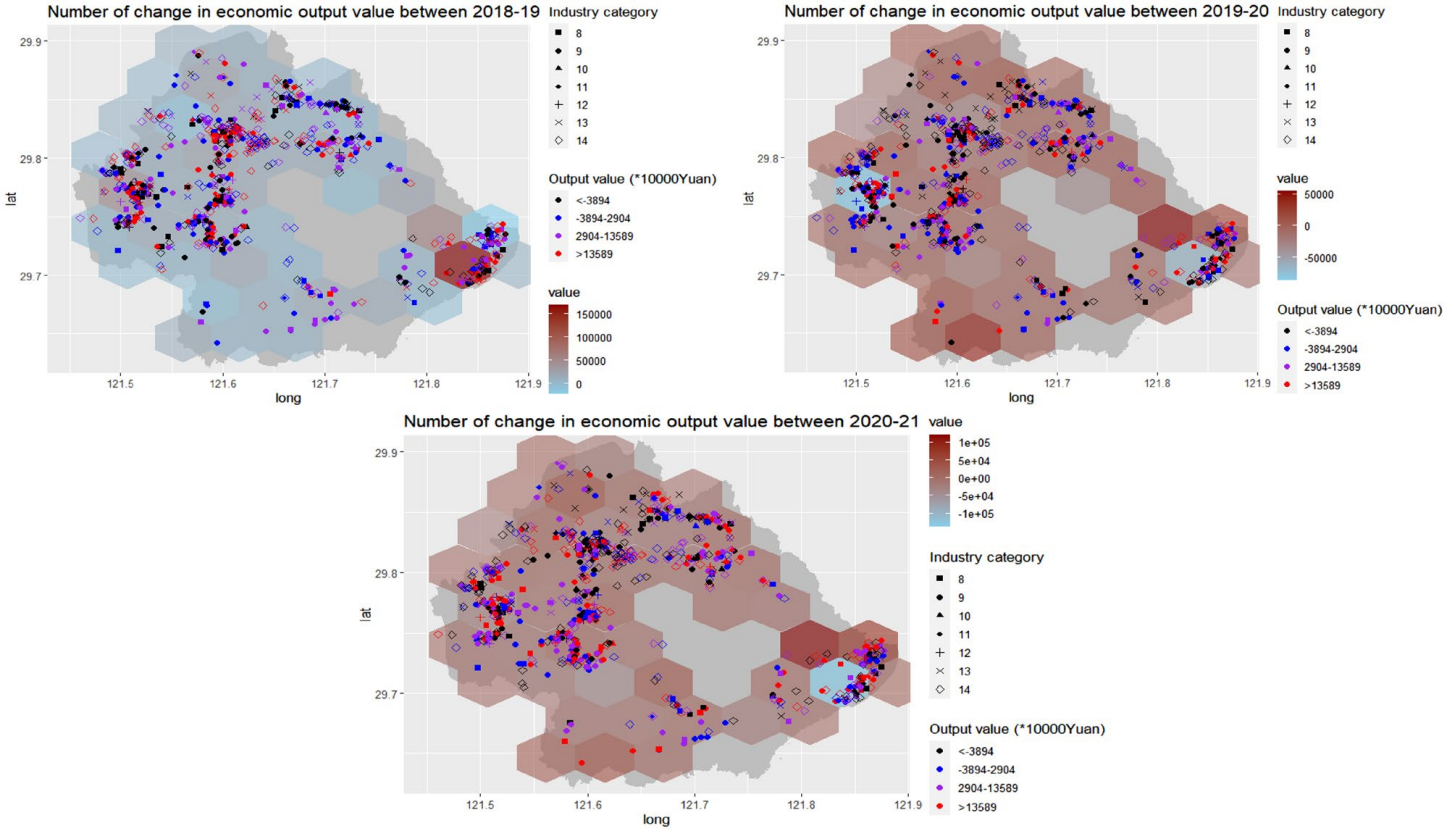
The distribution of economic output value dynamics in Yinzhou from 2018 to 2021 by space and time. (Industry category: 8 (motorcar), 9 (high-level equipment), 10 (new materials), 11 (intelligent home electrics), 12 (key fundamental part), 13 (fashion and clothes), 14 (new service and others)).

The mean difference in yearly economic output value was 18,214 (range: − 1,277,981 to 1,812,459) in Yinzhou between 2018 and 2019 (Fig. 2). The biggest increase in economic output value was found in the southeast area. For the period 2019–2020, the rise of the annual value was observed in the majority of regions, excluded in the west and southeast areas, with the mean difference of − 10,992 (range: − 7,379,274 to 458,454). From 2020 to 2021, the mean difference in yearly value was 2307 (range: − 2,406,953 to 2,567,025), and there was an obvious decrease in the southeast region for the period.

### Spatial cluster analysis over time

Spatial autocorrelation analysis was performed for all independent variable and economic dynamics during the study period before developing GWR model. Moran's I was used in the study as the spatial cluster analysis tool. The results are shown in the Table 2.

**Table 2:** Moran's I statistical results for spatio-temporal cluster analysis.

Year	Variable	Moran's I	Z-score	P-value
2018	The annual economic output value	0.67	4.23	0.04
	The change in annual economic output value	0.54	6.67	0.03
	The annual number of positive online news	0.71	3.81	0.02
	The percentage of positive online news	0.49	2.92	0.02
2019	The annual economic output value	0.78	4.83	0.02
	The change in annual economic output value	0.44	6.97	0.03
	The annual number of positive online news	0.78	3.71	0.01
	The percentage of positive online news	0.42	2.76	0.03
2020	The annual economic output value	0.60	4.49	0.03
	The change in annual economic output value	0.50	6.81	0.01
	The annual number of positive online news	0.77	3.72	0.01
	The percentage of positive online news	0.44	2.74	0.03
2021	The annual economic output value	0.71	4.80	0.02
	The change in annual economic output value	0.59	6.72	0.01
	The annual number of positive online news	0.67	3.88	0.03
	The percentage of positive online news	0.46	2.86	0.01

Spatial autocorrelation analysis showed that the values of Moran’s I of the annual economic output value, the change in the value between years, the yearly number of positive online news and the percentage of positive online news were both positive (Moran’s I range: 0–1), with the significant statistical level. The results indicated that all the variables showed cluster characteristics in space. This can be seen as the foundation for developing GWR model, and also provided the prerequisite for the validation of the model.

### Modeling online news with economic dynamics by GLM

The GLMs combining the yearly number of positive online news and the percentage of positive online news have good performance in predicting the economic dynamics, when consider the effect of industry policy. The goodness-of-fit of the models demonstrated in Table 3. The results indicated that the annual positive online news and the percentage of positive online news have positive contribution to the economic dynamics with the statistical significance (P < 0.05) (Table 3).

**Table 3:** GLM estimation results and goodness-of-fit.

Prediction	Variable	Estimate	Std. Error	T-value	P-value
Economic output value	The annual positive online news	50.86	0.09	33.34	0.02
	The percentage of positive online news	41.35	0.03	35.71	0.01
R^2^ = 0.77, AIC = 2030.06					
Change in economic output value	The annual positive online news	38.49	0.06	21.81	0.03
	The percentage of positive online news	46.61	0.05	19.60	0.03
R^2^ = 0.72, AIC = 1285.59					

In the GLM of predicting economic output value, with each unit of the annual number of positive online news growing, the value of annual economic output value increased by 50.86 units. 1 unit of the percentage of positive online news rising, the value can increase by 41.35 units. Moreover, in the GLM of forecasting change in the economic output value, the change value of annual economic output value climbed by 38.49 units, when the annual positive online news increased each unit. Similarly, the change value raised by 46.61 units, when the percentage of positive online news increased 1 unit.

### Estimating economic dynamics using online news by GWR

Firstly, we determined the optimal bandwidth by the smallest AIC value and standard residual in the model for each year. As a result, the optimal bandwidths ranged from 115.67 to 121.32 for the study period. The GWR model showed that the value of local coefficients for each predictor varies from area to area, with significant differences in the minimum, maximum and mean coefficients (Table 4).

**Table 4:** Estimation of trained GWR model for each variable and goodness-of–fit for model training period.

Prediction	Variable	2018			2019		
		Min	Mean	Max	Min	Mean	Max
Economic output value	The annual number of positive online news	− 23.34	27.89	78.43	− 16.25	23.07	68.26
	The percentage of positive online news	− 18.60	15.93	46.87	− 13.90	11.76	38.81
		R^2^ = 0.82, AIC = 156.23			R^2^ = 0.83, AIC = 137.71		

		2018–2019			2019–2020		
Change in economic output value	The annual number of positive online news	− 13.24	16.58	46.64	− 18.58	17.76	48.38
	The percentage of positive online news	− 18.16	19.22	52.98	− 22.92	24.66	71.72
		R^2^ = 0.78, AIC = 85.01			R^2^ = 0.80, AIC = 72.53		

For the model training period (2018–2019), the goodness-of-fit of the developed GWR models were demonstrated by the R^2^ and AIC values. For instance, the R^2^ value of the model for predicting economic output value in 2018 was 0.82. This indicated that the model could explain 82% of the change in the yearly economic output value (Table 4).

According to the R^2^ and AIC values, it could be found that the goodness-of-fit of the GWR model generally is better than GLM. GWR has greater capacity to estimate the spatio-temporal patterns of economic dynamics. This showed the advantages of the GWR model, which estimates the local regression coefficient by spatial unit. As a result, the model could fit the contribution of each predictor for each area separately^22^.

**Figure 3:**
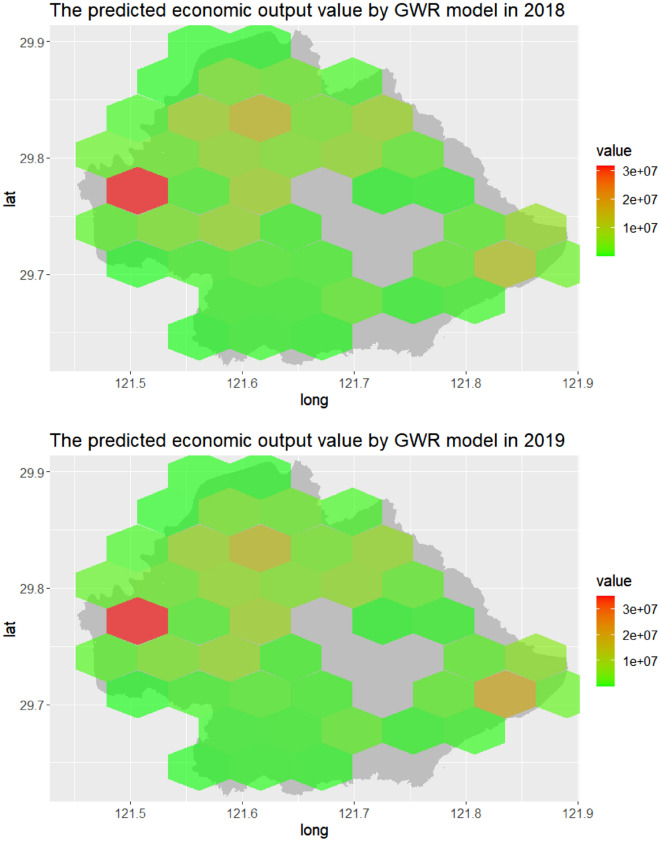
The spatial distribution of the annual economic output value based on the number of yearly positive online news using trained GWR model in Yinzhou, 2018–2019.

**Figure 4:**
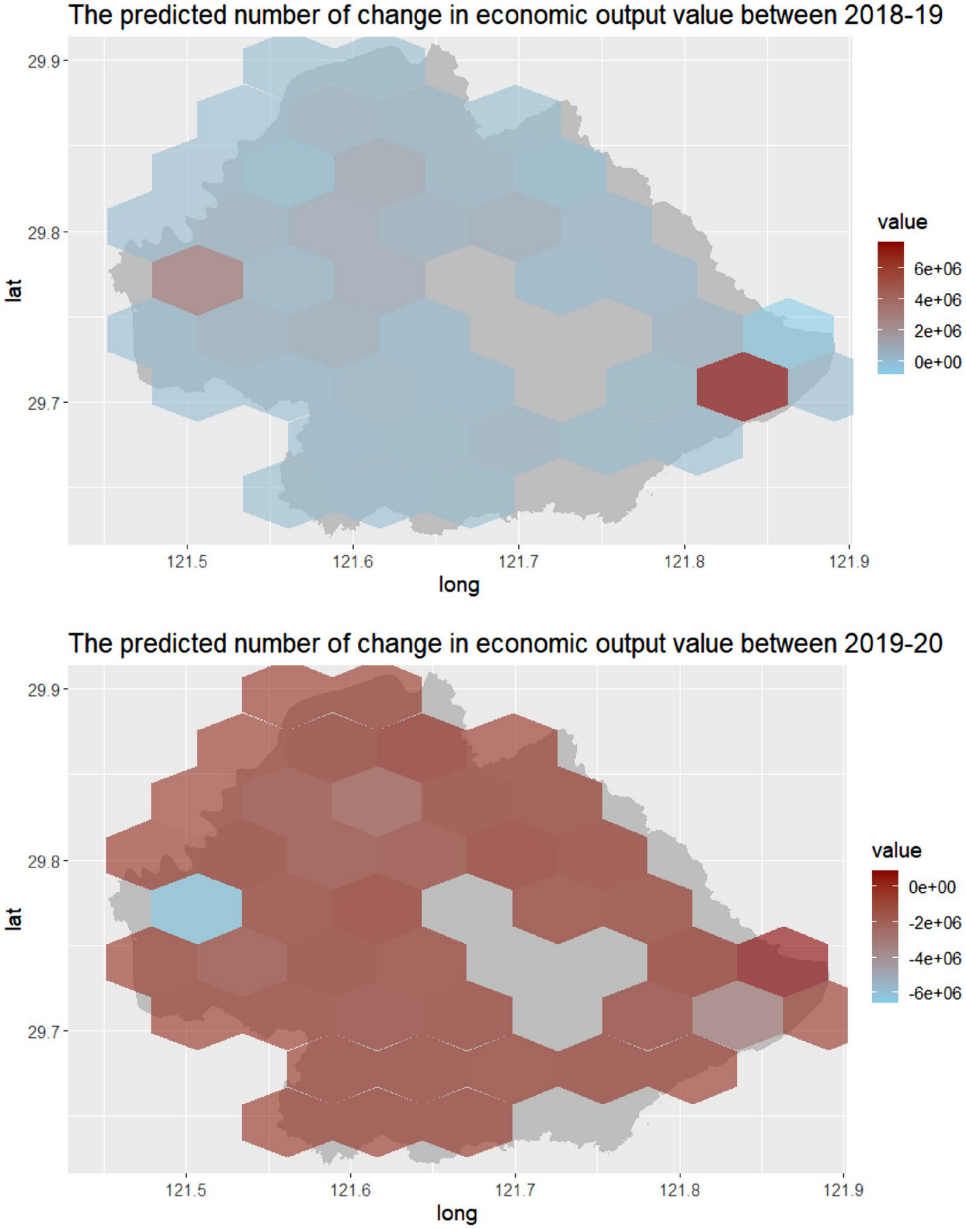
The spatial distribution of the yearly change in economic output value based on the percentage of yearly positive online news using trained GWR model in Yinzhou, 2018–2019.

Additionally, it seemed that the annual number of positive online news could better predict the economic output value by the GWR model, with relatively higher regression coefficient in each year. Similarly, the percentage of positive online news had a greater potential in the forecasting of change in economic output value by the GWR model for each year. Figure 3 showed the spatial distribution of the annual economic output value based on the number of yearly positive online news using trained GWR model from 2018 to 2019. Similarly, Fig. 4 demonstrated the annual change in economic output value using the percentage of the news by trained GWR model for the model training period (2018–2019).

**Figure 5:**
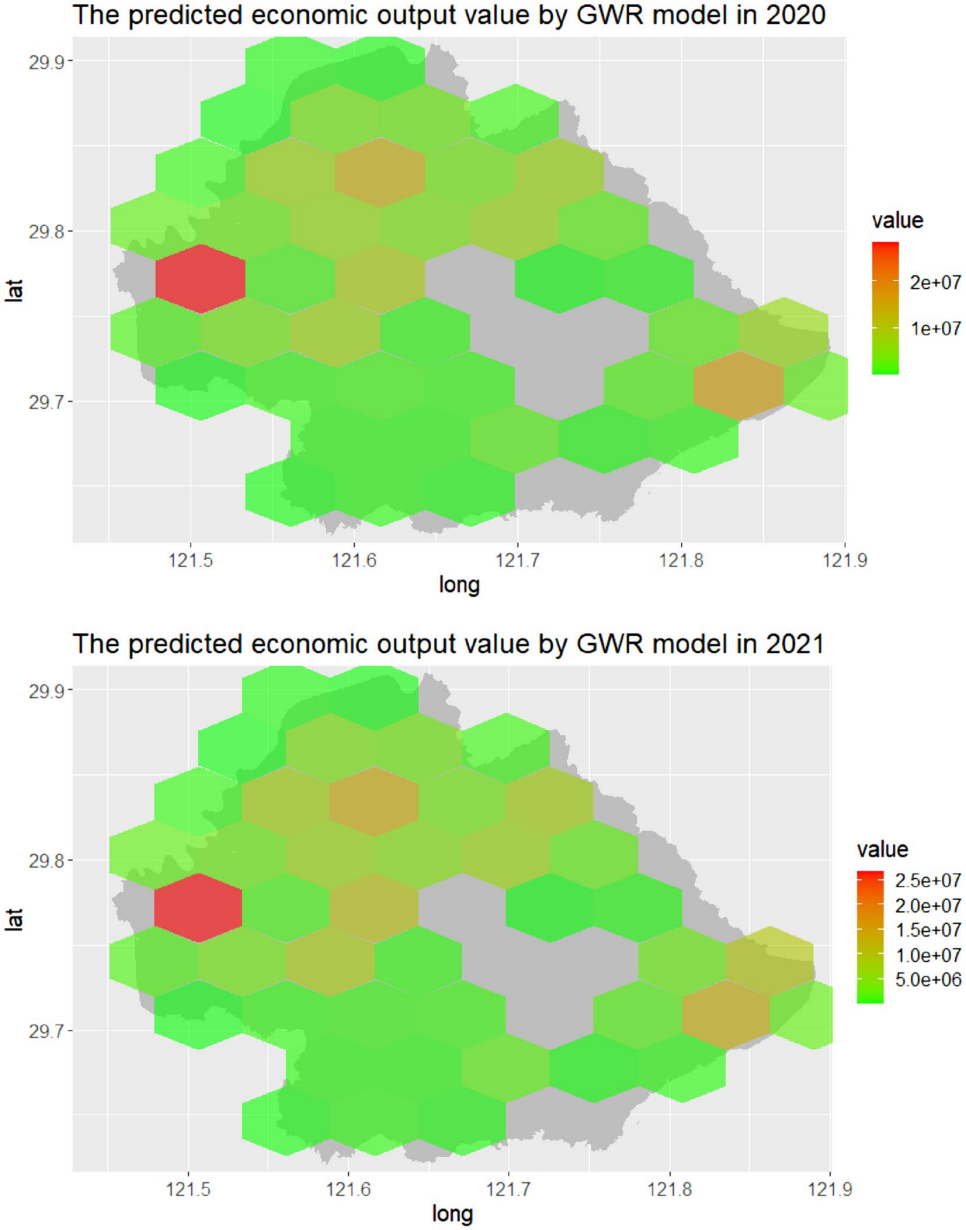
The spatial distribution of the predicted annual economic output value based on the number of yearly positive online news by GWR model in Yinzhou, 2020–2021.

**Figure 6:**
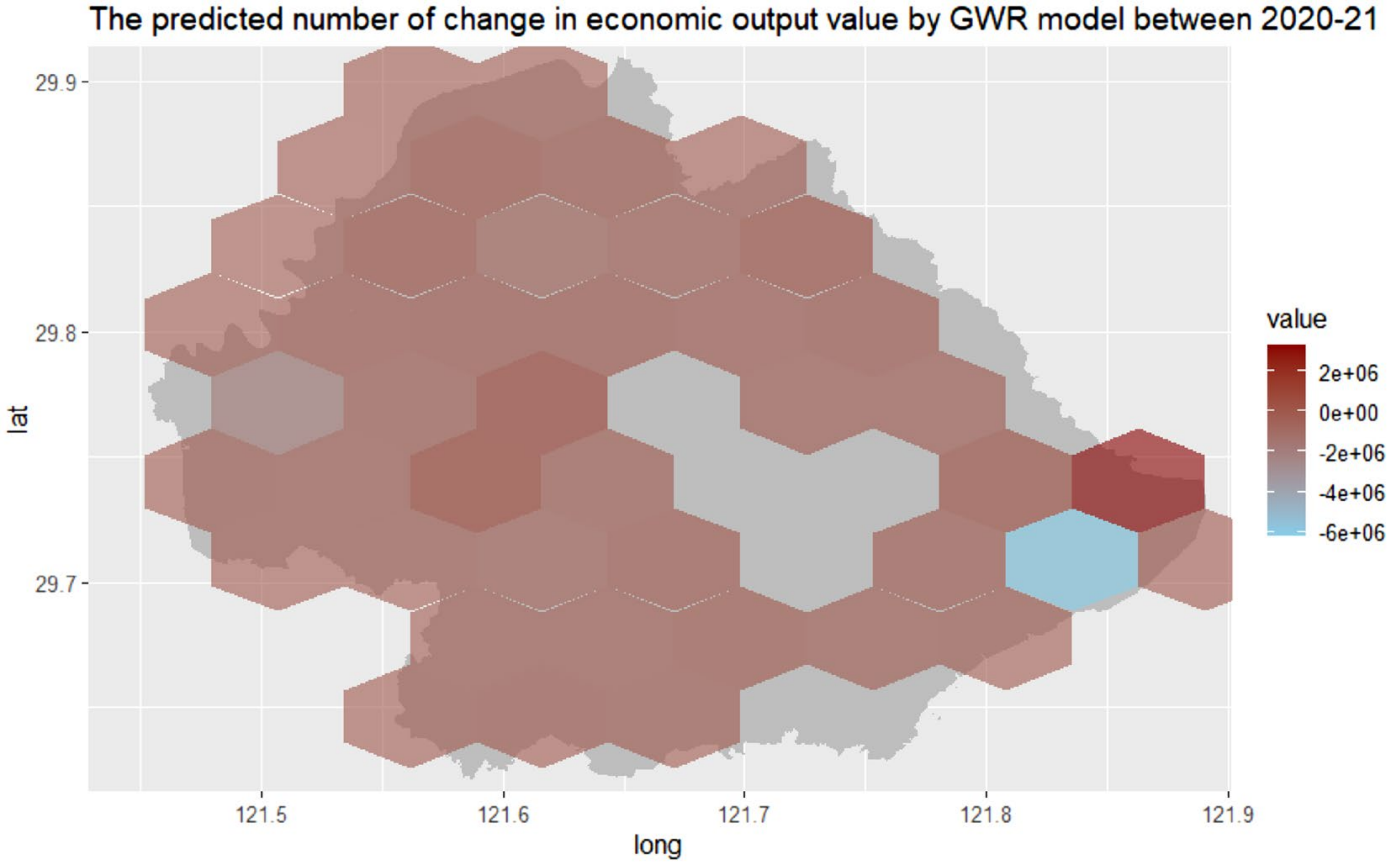
The spatial distribution of the predicted yearly change in economic output value based on the percentage of yearly positive online news by GWR model in Yinzhou, 2020–2021.

Then, we predicted the yearly economic output value and annual change in economic output value using the yearly number of positive online news and the percentage of the news separately for the model predictive period (2020–2021). Figure 5 showed the spatial distribution of the predicted annual economic output value based on the number of yearly positive online news from 2020 to 2021. In addition, Fig. 6 demonstrated the spatio-temporal distribution of the predicted yearly change in economic output value based on the percentage of yearly positive online news for the model predictive period (2020–2021).

The evaluation of forecasting performance of the predictive models is presented in Table 5. The table shows that the predictive capacity of the developed GWR models is performed well with high Pearson correlations (the yearly economic output value in 2020: 0.96, the yearly economic output value in 2021: 0.94, the annual change in economic output value between 2020 and 2021 = 0.97, p < 0.01). The predictive GWR models were also robust as showed by the low values of the MAPE in economic dynamics forecasting (the yearly economic output value in 2020: 1.26, the yearly economic output value in 2021: 1.94, the annual change in economic output value between 2020 and 2021 = 1.77), which measures the discrepancies between the off-target model predictive economic dynamics and the observed values for the model predictive period (2020–2021).

**Table 5:** Similarity metrics between the observed and predicted economic dynamics using developed GWR models.

	Pearson correlation	RMSE	MAPE
The predicted annual economic output value in 2020	0.96*	781.26	1.26
The predicted annual economic output value in 2021	0.94*	859.74	1.94
The predicted annual change in economic output value between 2020 and 2021	0.97*	349.12	1.77

*P < 0.01.

## Discussion

Economic output value is a dynamic process with the spatio-temporal patterns. Compared with the simple time model, the spatio-temporal model constructed from the two dimensions of time and space can predict and warn the time range and geographical area most likely to fluctuate in the future, and better provide theoretical and scientific basis for early warning and predictions^19^. In recent years, the rapid development of spatial information technology combines spatial analysis and visual expression methods, which can intuitively reflect the spatial and temporal distribution and change characteristics of a variety of subjects, and provide technical support for big data and multi-dimensional information in economy in space and time^20^.

Kulldorff et al.^21,22^ proposed prospective space–time scan statistics and prospective space–time permutation scan statistics in 2001 and 2005. The two methods do not need to limit the size, location and scale of aggregation before analysis. As an exploratory analysis, they can fully mine the data information and find the abnormal spatio-temporal aggregation of variables^23^. Spatiotemporal scanning statistics often use geometric (circular, elliptical or square) scanning windows to find clusters, which is not suitable when the occurrence of unusual values tends to gather in irregular areas. The maximum linkage space–time permutation scan statistics and co-clustering approach proposed in recent years are not limited by the scanning shape and size. They can quickly detect unusual patterns with irregular geometric regions and provide more details about the spatial and temporal range^24,25^.

The key to better monitor economic dynamics is whether the monitoring system can send out alerts quickly and accurately. Therefore, intuitive and reliable risk estimation methods can help governments respond to the upcoming fluctuations in time. With the rapid growth and improvement of electronic "big data" information of enterprises, the methods of monitoring and early warning in economic dynamics are being continuously improved.

Although there are still deficiencies in economic dynamics predictions based on "big data", this does not deny the potential value of "big data" for economic dynamics forecasting^26^. Improving the ability of data collection, management, analysis and application, and making "big data" help the prediction of economic dynamics is the key issue at present. Facing the "big change" led by "big data", with the continuous construction of economic "big data" system, the increasing coverage and reliability of economic monitoring system, the further popularization of Internet and mobile devices, and the continuous innovation and development of data integration and mining technology, based on traditional economic monitoring data, integrate data sources such as Internet, geography, transport and energy consumption in the future, dynamic multi-dimensional perspective analysis and display will be the research direction in this field to provide more and more comprehensive economic and enterprises information and improve the accuracy and timeliness of economic dynamics prediction and early warning.

## Conclusion

This study explored the spatio-temporal patterns of economic output values in Yinzhou, Ningbo, China, and proposed GLM and GWR models to predict the dynamics. The use of online news has a great potential to predict such dynamics, with better performance in the GWR model. This can be seen as novel data source in the future forecast. The advanced spatio-temporal analysis enables governments to garner insights about the patterns of economic dynamics over time. This may enhance the ability of government to formulate economic plans and to predict the implementation of the plan. The proposed model may be extended to greater geographic area to validate such approach.

## Data Availability

The datasets generated during and/or analysed during the current study are available from the corresponding author on reasonable request.
